# Rapid strawberry domestication left room to grow

**DOI:** 10.1093/plcell/koae015

**Published:** 2024-02-03

**Authors:** Andrew C Willoughby

**Affiliations:** Assistant Features Editor, The Plant Cell, American Society of Plant Biologists; Department of Biology, University of North Carolina at Chapel Hill, Chapel Hill, NC 27514, USA

The domestication of strawberries (*Fragaria x ananassa*) stands as an example of remarkable agricultural advancement, with the original hybrids arising less than 300 years ago (Liston et al. 2014). Contrary to what one might expect from a speedy and recent domestication process starting from small founder populations, cultivated strawberries exhibit substantial genetic diversity, attributable to subsequent hybridization events (Hardigan et al. 2021).

In their new study, **Zhen Fan and Vance M. Whitaker** (Fan and Whitaker 2024) analyze 289 strawberry genomes from wild species, heirloom cultivars, and modern varieties from University of Florida (UF) and University of California at Davis (UCD) breeding programs. Their research traces genomic changes throughout strawberry domestication and identifies differing selection pressures in the UF and UCD cultivars. Through a combination of identifying the genomic regions under both natural and artificial selection in strawberry, as well as highlighting loci not yet under selection that control agriculturally important traits, the work of **Fan and Whitaker** provides a genomic roadmap for future improvement of strawberry.

Cultivated strawberry initially combined the larger fruits of *F. chiloensis* subsp. *chiloensis* and the resilience of *F. virginiana* subsp. *virginiana*, but the authors find that selection has progressively increased the relative proportion of *F. virginiana* ancestry (see Fig.). Although the global admixture composition in the modern UF and UCD varieties is largely the same, chromosomal variation in proportion of species ancestry between these groups is common, hinting at differential selection for the subtropical climate at UF and the Mediterranean climate of UCD.

**Figure.**
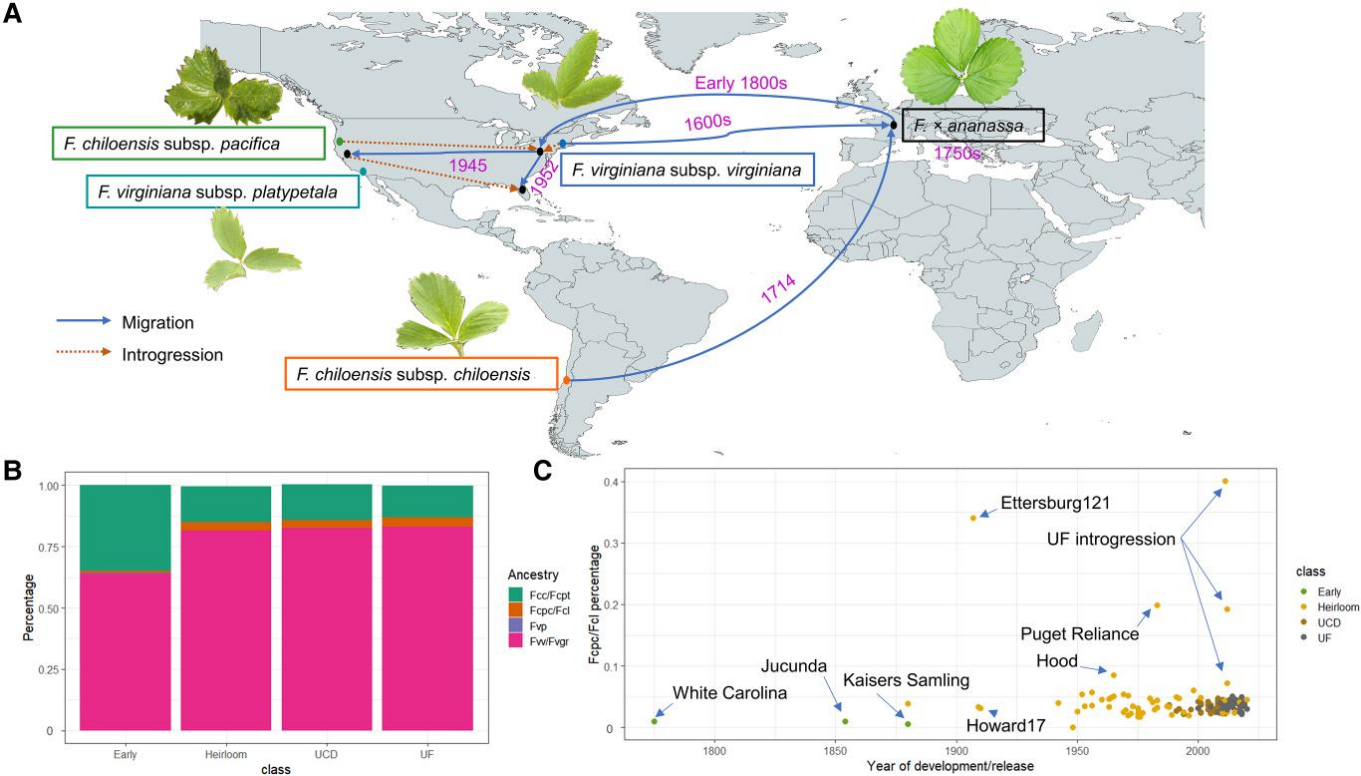
Strawberry domestication. Domestication began with the *F. x ananassa* hybrid between *F. virginiana* subsp. *virginiana* (Fvv/Fvgr) and *F. chiloensis* subsp. *chiloensis* (Fcc/Fcpt) less than 300 years ago (**A**), and progressively increased in the percentage of *F. virginiana* subsp. *virginiana* ancestry, stabilizing in the mid-1900s (**B**). Contributions by *F. chiloensis* subsp. *pacficia* and *F. chiloensis* subsp *lucida* remained constant after the introduction of *F. x ananassa* back to North America following additional hybridization events (**C**). Reprinted from Fan and Whitaker (2024), Figure 3.

In this study, the proportion of genomic regions found to be under selection in cultivated strawberries is larger than previous estimates (Hardigan et al. 2021) and larger than other crops, consistent with the short domestication history of strawberries (Fan and Whitaker 2024). The authors identify regions under selection unique to the modern breeding programs and find evidence that artificial selection is what drove the change in relative ancestry in cultivated strawberry to predominantly *F. virginiana*. The large numbers of wild samples allowed the authors to compare artificial selection during domestication to the effects of natural selection; they found regions of overlap that they hypothesize are related to climate adaptation.

**Fan and Whitaker** found that although genetic diversity has decreased in systematic breeding programs, more than half of the loci linked to fruit size and yield by a large genome wide association study (GWAS) are not currently under selection. These loci therefore represent untapped variation in domesticated strawberry that could improve future cultivars.
